# Mixed Autoimmune Hemolytic Anemia as the Initial Manifestation of Systemic Lupus Erythematosus: A Case Report

**DOI:** 10.7759/cureus.116935

**Published:** 2026-09-25

**Authors:** Tariq Iqbal, Cleopatra Chivero, Sharmin Farhana

**Affiliations:** 1 Acute Medicine, The Princess Alexandra Hospital NHS Trust, Harlow, GBR

**Keywords:** autoantibodies, direct antiglobulin test (dat), lupus nephritis, mixed autoimmune hemolytic anemia, systemic lupus erythematosus

## Abstract

Mixed autoimmune hemolytic anemia (mAIHA) is a rare subtype of autoimmune hemolytic anemia (AIHA) characterized by the coexistence of warm (immunoglobulin G (IgG)) and cold (immunoglobulin M (IgM)) autoantibodies. It may occur as a primary disorder or secondary to underlying conditions, including systemic lupus erythematosus (SLE). We report the case of a 16-year-old female who presented with severe hemolytic anemia as the initial manifestation of SLE. On admission, her hemoglobin was 34 g/L with marked reticulocytosis and a positive direct antiglobulin test (DAT) demonstrating both IgG and IgM antibodies, confirming mAIHA. Immunological investigations revealed positive antinuclear antibody (ANA), anti-double-stranded DNA (anti-dsDNA) antibody, and anti-Smith antibody, supporting a diagnosis of SLE. At the time of the first presentation, she had no other clinical features suggestive of SLE. She was treated with blood transfusions and corticosteroids, resulting in significant improvement, and was discharged on a tapering prednisolone regimen. Shortly after, she was readmitted with a malar rash, arthralgia, joint swelling, nephrotic-range proteinuria, and worsening renal function. She was subsequently diagnosed with SLE complicated by lupus nephritis and recurrent AIHA. Following the rheumatology and nephrology review, she received intravenous methylprednisolone and was transferred to a tertiary care center for renal biopsy and further management. Renal biopsy demonstrated focal segmental glomerulosclerosis (FSGS). Further treatment with prednisolone, mycophenolate mofetil, intravenous belimumab, and hydroxychloroquine resulted in an excellent clinical response, with follow-up confirming remission. This case highlights mAIHA as a rare initial presentation of SLE and underscores the importance of early recognition, close monitoring, and multidisciplinary management, as patients presenting with AIHA may be at increased risk of developing lupus nephritis and other severe SLE-related complications.

## Introduction

Mixed autoimmune hemolytic anemia (mAIHA) is a rare subtype of autoimmune hemolytic anemia (AIHA) characterized by the coexistence of warm and cold autoantibodies that cause hemolysis [1,2]. Hemolysis is predominantly extravascular, occurring in the spleen and liver, although intravascular hemolysis can also occur. Diagnosis is established with a direct antiglobulin test (DAT), which detects immunoglobulin G (IgG) and immunoglobulin M (IgM) autoantibodies. Warm IgG autoantibodies bind to red blood cells at 37°C, while cold IgM autoantibodies react optimally at 4°C but may also react at temperatures ranging from 4°C to 34°C [1].

AIHA may be classified as primary or secondary. Approximately 50% of AIHA cases have no identifiable underlying cause and are therefore classified as primary, or idiopathic, AIHA [2]. Secondary AIHA is associated with autoimmune diseases such as systemic lupus erythematosus (SLE), bacterial and viral infections, malignancies such as lymphoma, and certain medications. Less than 10% of AIHA is mAIHA. Up to 10% of SLE patients have AIHA. Moreover, mAIHA presenting as an initial manifestation of SLE is quite rare [2].

We present a case of mAIHA in a patient who initially presented with severe anemia requiring a blood transfusion. Difficulty cross-matching compatible blood prompted further investigations, and the DAT was positive for warm and cold autoantibodies, making compatible blood difficult to obtain. To minimize further hemolysis during transfusion, special precautions included the use of a blood warmer and corticosteroid coverage.

Further evaluation identified SLE as the underlying cause of her mAIHA, with mAIHA representing the initial manifestation of SLE, which, in the absence of any other clinical manifestations, underscores the rare and atypical early presentation of SLE in our case.

## Case presentation

First admission (February 2025)

A 16-year-old African female was admitted with a six-day history of fever, headache, backache, and tinnitus. She also reported a one-day history of nausea and vomiting. She denied cough, sore throat, diarrhea, urinary symptoms, rash, joint pain, joint swelling, hematemesis, melena, and menorrhagia.

On examination, her temperature was 37.7°C, blood pressure was 135/58 mmHg, heart rate was 100 beats/minute, and oxygen saturation was 98% on room air. She had marked pallor without signs of meningeal irritation, significant lymphadenopathy, clubbing, cyanosis, or clinically apparent jaundice. Cardiovascular examination revealed a systolic flow murmur. The remainder of the systemic examination was unremarkable. With the above history and examination, the initial diagnosis was meningitis, although there were no signs of meningeal irritation. Another diagnosis was severe anemia based on clinical examination.

Laboratory investigations revealed a hemoglobin level of 34 g/L and a mean corpuscular volume of 114 fL (Table 1). White blood cell and platelet counts were within the normal range. Blood gases did not show any lactic acidosis. A peripheral blood smear demonstrated severe anemia with anisocytosis, polychromasia, poikilocytosis, teardrop cells, and stomatocytes. Serum urea, electrolytes, vitamin B12, folate, and thyroid-stimulating hormone levels were within the normal range. Chest radiography showed no evidence of consolidation or pulmonary infiltrates (Figure 1). The CT head scan was normal, while the ECG showed sinus rhythm (heart rate 100/minute) (Video 1, Figure 2).

**Table 1:** Hematological laboratory findings Laboratory results obtained from the patient’s NHS medical records. FBC: full blood count; Hb: hemoglobin; WBC: white blood cell count; MCV: mean corpuscular volume; RBC: red blood cell count; PCV: packed cell volume (hematocrit); MCH: mean corpuscular hemoglobin; NRBCs: nucleated red blood cells

Laboratory Test	Result	Unit	Normal Values
Hb	34	g/L	115–165
WBC	4.4	x10^9^/L	4.0–11.0
Platelets	200	x10^9^/L	150–450
MCV	114	fL	76–96
RBC	1.05	x10^12^/L	3.80–4.80
PCV	0.120	L/L	0.370–0.470
MCH	32.4	pg	27.0–32.0
Neutrophils	2.6	x10^9^/L	2.0–8.0
Lymphocytes	1.5	x10^9^/L	1.0–4.8
Monocytes	0.3	x10^9^/L	0.2–0.8
Eosinophils	0.0	x10^9^/L	0.0–0.4
NRBCs	0.2	x10^9^/L	0.0
Reticulocytes %	11.3	%	0.2–2.0
Absolute reticulocyte count	0.111	x10^12^/L	0.020–0.130

**Figure 1:**
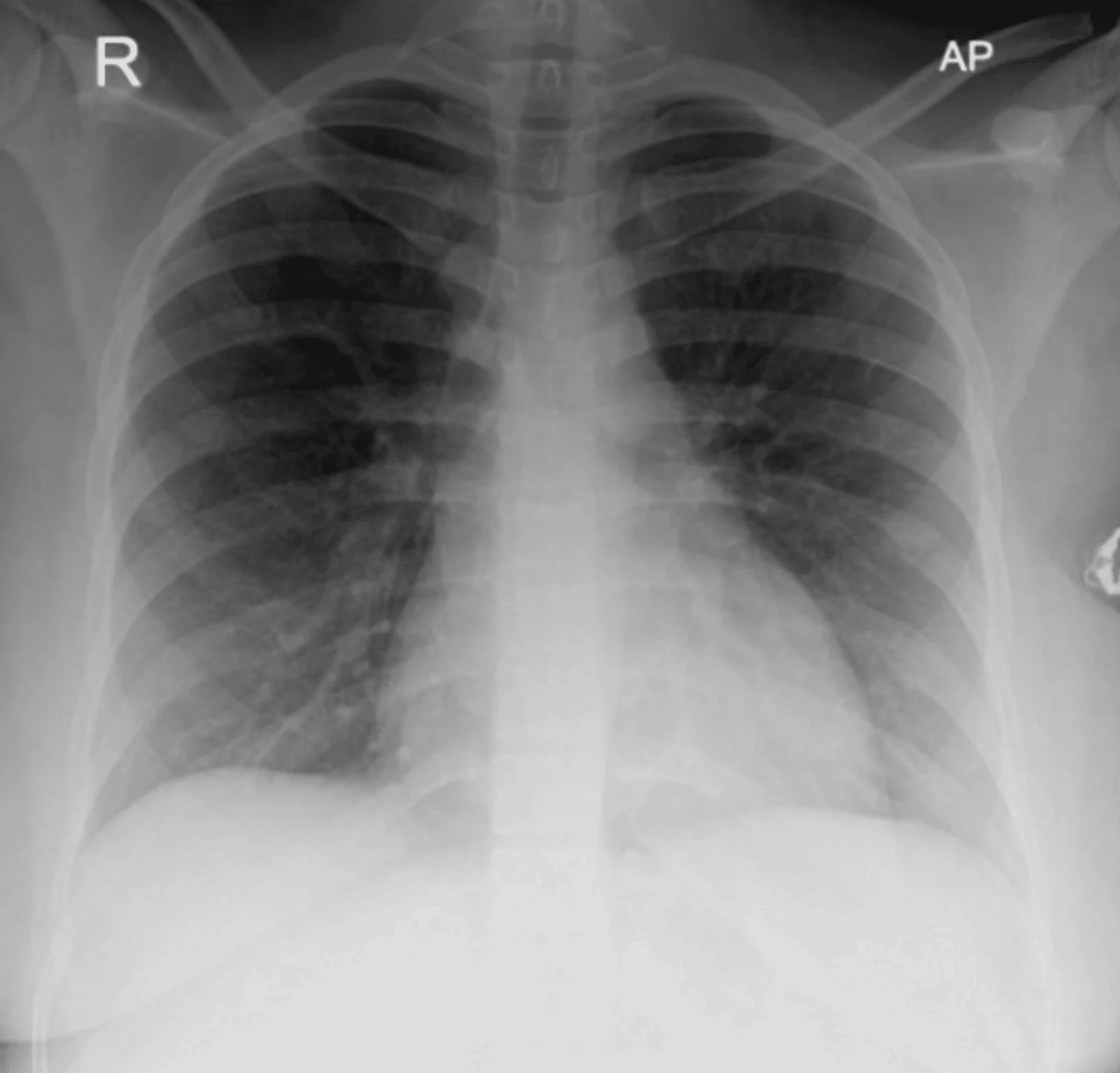
Chest X-ray findings This chest X-ray was taken in February. There were no abnormal findings.

**Video 1:** CT head findings CT head scan taken in February was normal.

**Figure 2:**
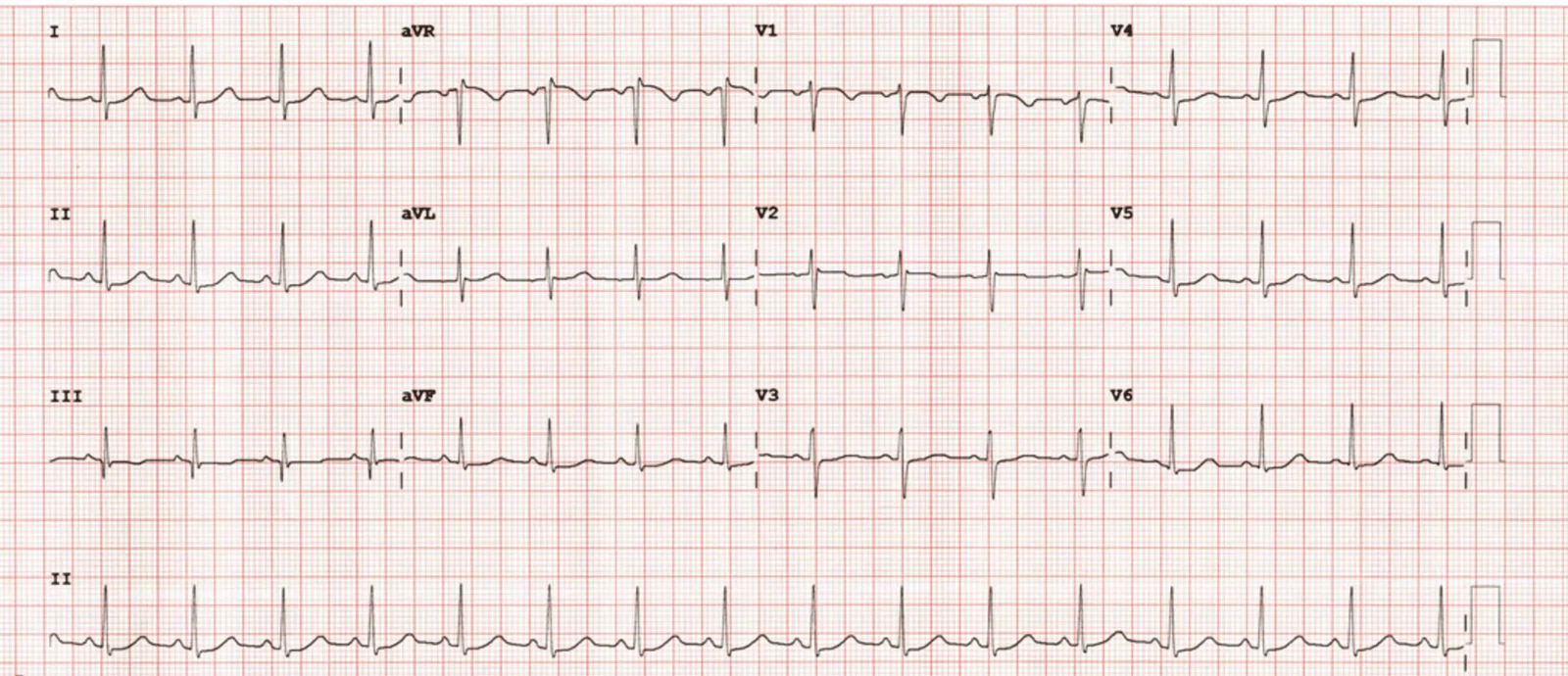
ECG findings This ECG was taken in February. It shows sinus rhythm.

Initial laboratory investigations confirmed anemia with reticulocytosis, elevated lactate dehydrogenase (LDH), and macrocytosis, consistent with hemolytic anemia. Moreover, her liver function tests (LFTs) showed mildly raised bilirubin (Table 2). Given the patient's ethnic background, hemoglobinopathy was initially considered but was excluded following further hematological investigations. Direct and indirect antiglobulin (Coombs) tests subsequently confirmed mAIHA (Table 3). Secondary causes, including infection, malignancy, and drug-induced hemolysis, were excluded through appropriate investigations. An autoimmune screen, including testing for SLE, was performed during the initial admission; however, the results remained pending at the time of discharge and became available only during the subsequent admission, ultimately identifying SLE as the underlying etiology of the patient's mAIHA.

**Table 2:** Laboratory results ALT: alanine aminotransferase; CK: creatine kinase; LDH: lactate dehydrogenase

Laboratory Test	Result	Unit	Normal Values
Total bilirubin	25	µmol/L	0–21
Direct (conjugated) bilirubin	10	µmol/L	0–9
ALT	25	IU/L	0–34
Creatine kinase/CK	162	IU/L	25–200
LDH	724	IU/L	125–220
Sodium	134	mmol/L	133–146
Potassium	4.2	mmol/L	3.5–5.3
Creatinine	51	µmol/L	53–97
Urea	2.2	mmol/L	2.5–7.8

**Table 3:** Direct antiglobulin Coombs test results DAT: direct antiglobulin test; AHG: anti-human globulin; IgG: immunoglobulin G; IgA: immunoglobulin A; IgM: immunoglobulin M; C3c: complement component 3c; C3d: complement component 3d; C4: complement component 4

Investigation	Findings	Reference Value / Control
Direct Antiglobulin Test (DAGT/DAT)	Positive	Negative
AHG	4+	Negative
IgG	3+	Negative
IgA	1+	Negative
IgM	2+	Negative
C3c	1+	Negative
C3d	4+	Negative
C4	3+	Negative

Treatment During First Admission

The patient was initially commenced on intravenous ceftriaxone 2 g twice daily for suspected meningitis. However, treatment was discontinued after 24 hours, as there was no clinical, biochemical, or radiological evidence to support the diagnosis, and inflammatory markers remained within normal limits. Lumbar puncture, which had initially been deferred because of profound anemia, was subsequently cancelled.

Following the availability of compatible blood products, the patient received a transfusion of three units of packed red blood cells (PRBCs) administered via a blood warmer. Given the diagnosis of AIHA, oral prednisolone tablets were initiated at a dose of 1 mg/kg/day (70 mg once daily) in accordance with hematology recommendations. Concurrent supportive therapy included oral tablet folic acid 5 mg once daily and oral tablet omeprazole 20 mg once daily.

Further investigations were undertaken to determine the underlying etiology of the AIHA, including an autoimmune screen; however, the results were pending at the time of discharge.

The patient demonstrated a favorable clinical and hematological response following PRBC transfusion and corticosteroid therapy. She was discharged on day 6 with a hemoglobin concentration of 114 g/L and significant symptomatic improvement. At discharge, she remained on prednisolone 70 mg once daily, folic acid 5 mg once daily, and omeprazole 20 mg once daily. A corticosteroid tapering regimen was planned, with prednisolone to be reduced by 5 mg weekly commencing two weeks after discharge.

Follow-up was arranged through the Acute Medicine Same Day Emergency Care (SDEC) service and subsequently with the hematology outpatient team. At review in March 2025, the patient remained clinically stable, with no recurrence of symptoms and persistently normal hemoglobin levels. Continued outpatient follow-up was scheduled at four- to five-week intervals.

Second admission (May 2025)

Unfortunately, she missed a hematology follow-up appointment. During her second admission, she presented with a five-day history of intermittent fever, diarrhea, vomiting, fatigue, and palpitations. Her mother reported intermittent joint pain, predominantly involving the hands. She also noted periorbital swelling.

On examination, the patient was febrile with a temperature of 38°C, a heart rate of 123 beats/minute, and an oxygen saturation of 98% on room air, while her blood pressure was normal. She had acrocyanosis of the fingers, swelling in her hands, periorbital edema, and a malar rash (Figures 3, 4).

**Figure 3:**
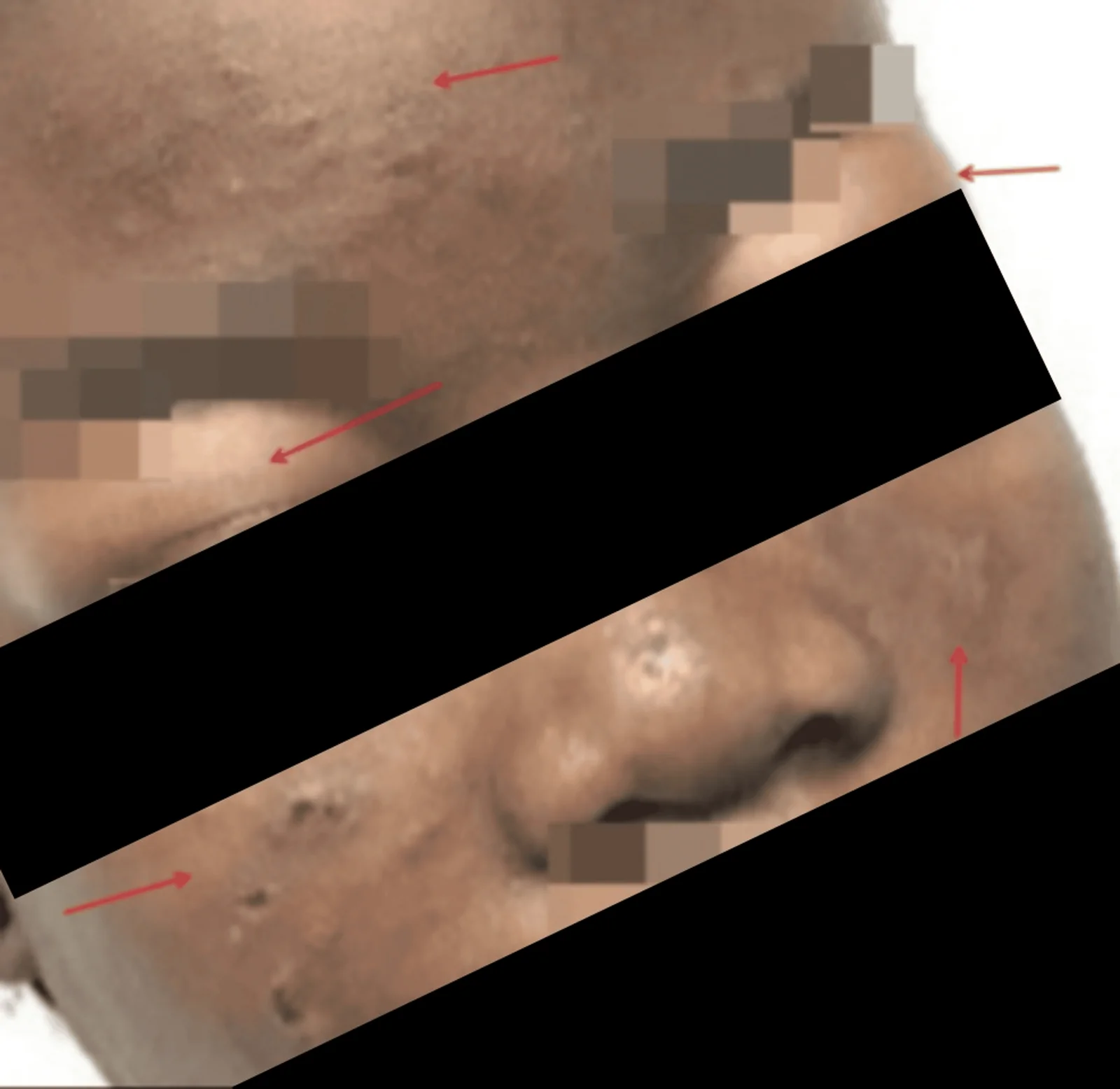
Malar rash and periorbital edema observed during the secondary admission of the patient Arrows pointing at the eyelids highlight the periorbital edema, while the ones pointing at the cheek showcase the malar rash. Photographs were obtained from the patient's NHS medical records with the consent of both the patient and the parent.

**Figure 4:**
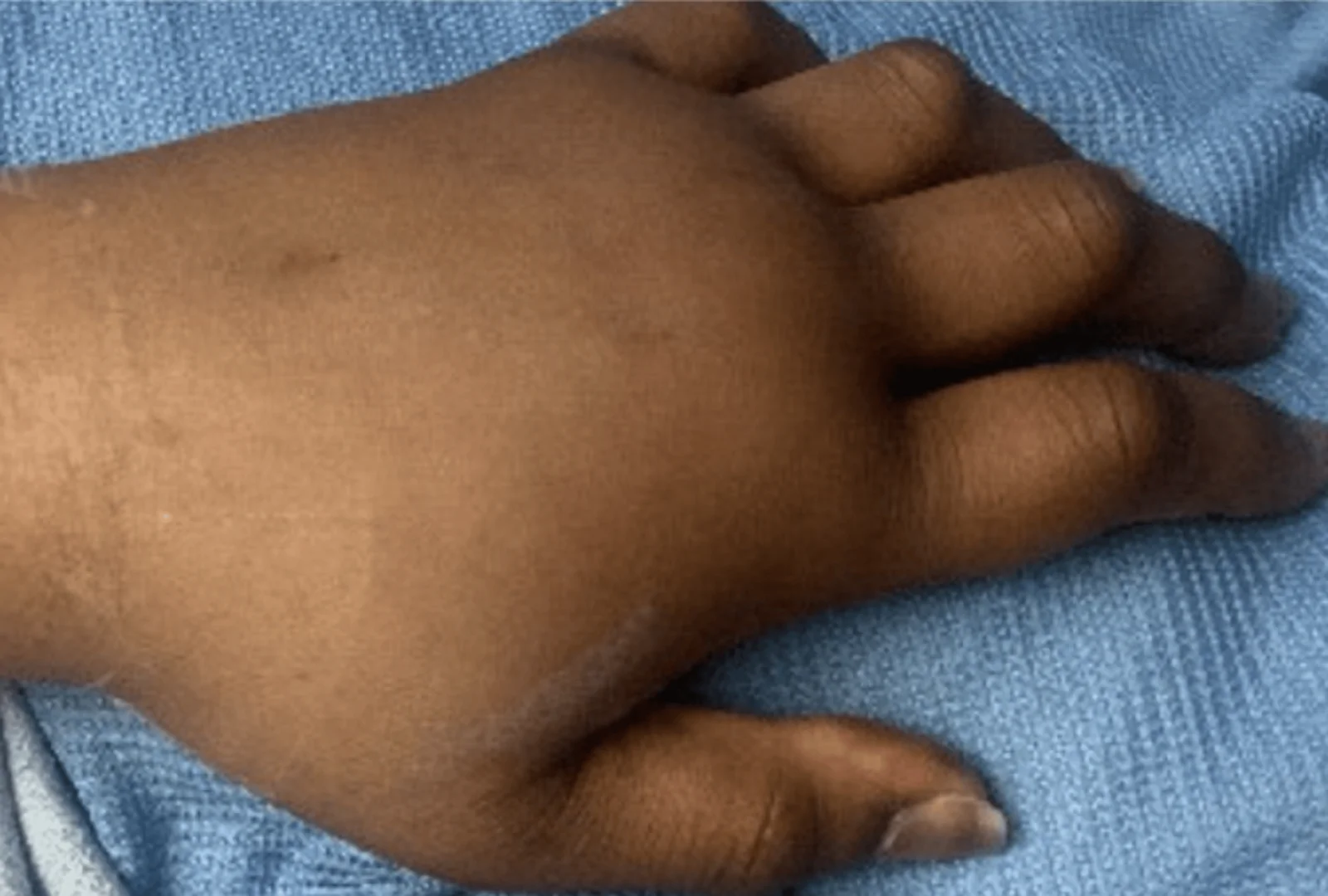
Swelling of the hand of the patient during the second admission Photographs were obtained from the patient's NHS medical records with the consent of both the patient and the parent.

She had pallor on examination of her conjunctiva. The rest of the physical examination was unremarkable. Based on the patient's history, clinical examination, and review of the available investigations, a relapse of mAIHA secondary to an underlying autoimmune disorder was suspected. Given her presenting gastrointestinal symptoms, infectious etiologies, including viral and bacterial gastroenteritis, were also considered as differential diagnoses.

The autoimmune investigations requested during the previous admission subsequently became available and revealed positive antinuclear antibody (ANA) and anti-double-stranded DNA (anti-dsDNA) antibodies (Table 4). Repeat laboratory investigations demonstrated a progressive decline in hemoglobin from 114 g/L at the time of discharge from the initial admission to 75 g/L on re-presentation, with a further decrease to 59 g/L during admission (Table 5), consistent with recurrent hemolysis.

**Table 4:** Autoimmune serology (immunology) results ANA: antinuclear antibody; ENA: extractable nuclear antigen; anti-U1 RNP: anti-U1 ribonucleoprotein antibody; anti-Sm: anti-Smith antibody; SS-A: Sjögren syndrome-related antigen A (Ro); SS-B: Sjögren syndrome-related antigen B (La); anti-Jo-1: anti-histidyl transfer RNA (tRNA) synthetase antibody; anti-Scl-70: anti-topoisomerase I antibody; anti-CENP: anti-centromere protein antibody; anti-dsDNA: anti-double-stranded DNA antibody; aCL: anticardiolipin; IgG: immunoglobulin G; IgM: immunoglobulin M; C3: complement component 3; C4: complement component 4

Laboratory Test	Result	Unit	Normal Value
anti-dsDNA antibody	75.0	IU/mL	< 10.0
aCL IgG antibody	15.3	GPLu/mL	0.0–12.0
aCL IgM antibody	8.60	MPLu/mL	0.00–9.40
C3	0.81	g/L	0.83–1.93
C4	0.13	g/L	0.15–0.57
ANA Screen	Positive	-	Negative
ENA (ex. nuc Ag) panel	Positive	-	Negative
Anti-U1 RNP antibody	Positive	-	Negative
Anti-Sm antibody	Positive	-	Negative
Anti-Ro (SS-A) antibody	Negative	-	Negative
Anti-La (SS-B) antibody	Negative	-	Negative
Anti-Jo-1 antibody	Negative	-	Negative
Anti-Sci 70 antibody	Negative	-	Negative
Anti-CENP antibody	Negative	-	Negative

**Table 5:** Full blood count (FBC) Hb: hemoglobin; WBC: white blood cell count; MCV: mean corpuscular volume; RBC: red blood cell count; PCV: packed cell volume (hematocrit); MCH: mean corpuscular hemoglobin

Laboratory Test	Result	Unit	Normal Values
Hb	75	g/L	115–165
Repeat Hb	59	g/L	115–165
WBC	5.1	x10^9^/L	4.0–11.0
Platelets	304	x10^9^/L	150–450
MCV	95	fL	76–96
RBC	2.18	x10^12^/L	3.80–4.80
PCV	0.208	L/L	0.370–0.470
MCH	34.4	pg	27.0–32.0
Neutrophils	3.1	x10^9^/L	2.0–8.0
Lymphocytes	1.7	x10^9^/L	1.0–4.8
Monocytes	0.2	x10^9^/L	0.2–0.8
Eosinophils	0.0	x10^9^/L	0.0–0.4
Haptoglobin	<0.1	g/l	0.3-2

The patient was admitted for further investigation and management. A comprehensive septic screen, including blood, urine, and stool cultures, was performed. Urinalysis demonstrated significant proteinuria (>300 mg/dL) with trace hematuria. Chest radiography showed no acute cardiopulmonary abnormality, whereas electrocardiography demonstrated sinus tachycardia (Figures 5, 6). Blood gas analysis revealed no evidence of lactic acidosis. Blood, urine, and stool cultures remained negative, and respiratory viral screening was also negative.

**Figure 5:**
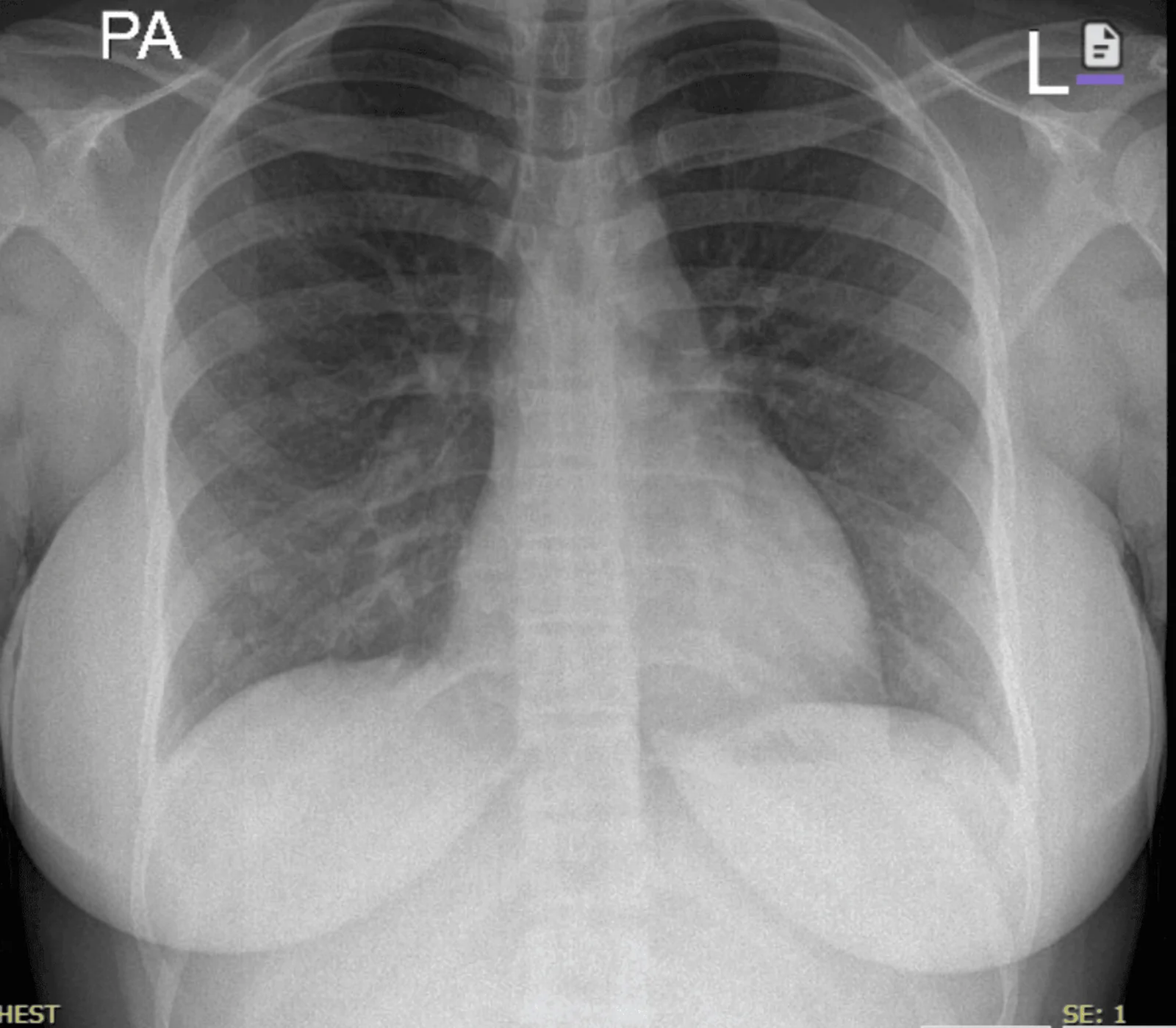
Chest X-ray findings This chest X-ray was taken in May. There were no abnormal findings.

**Figure 6:**
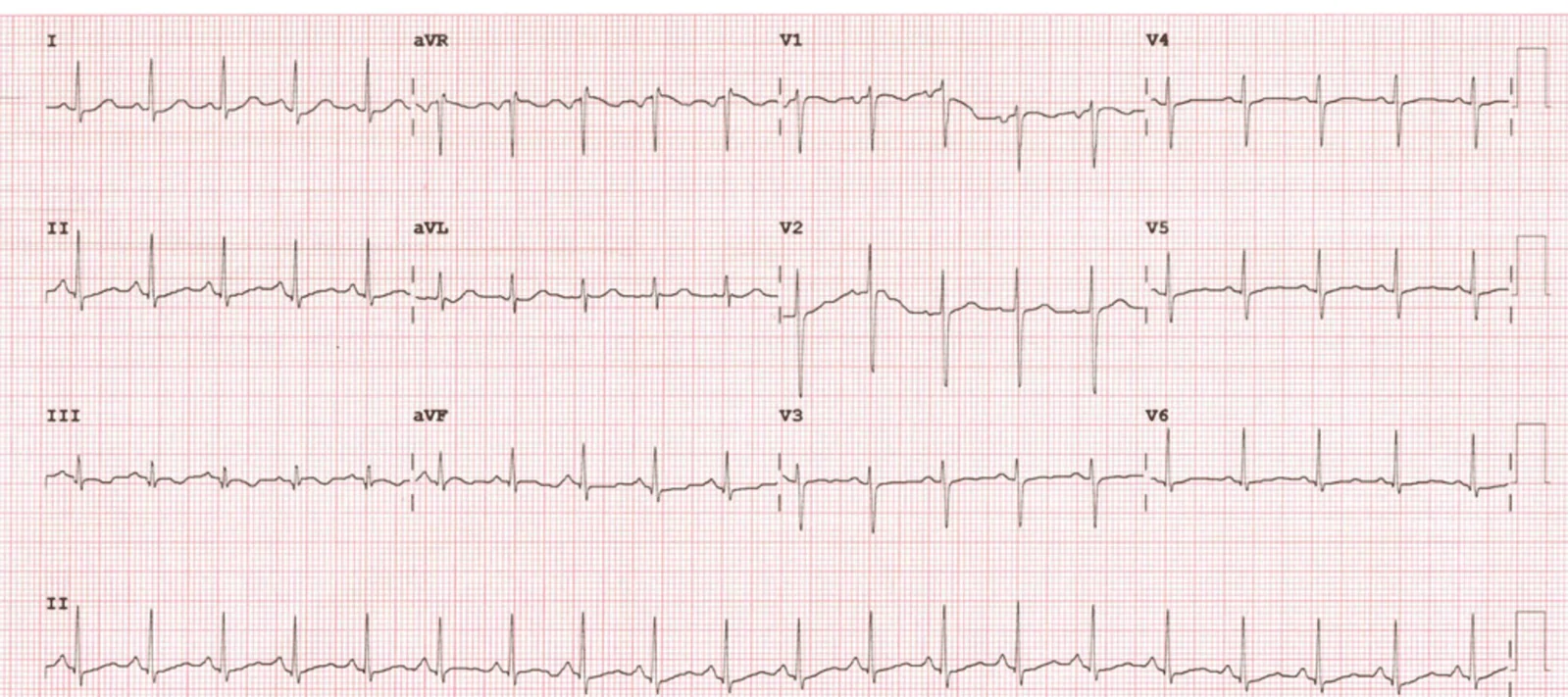
ECG findings This ECG was taken in May. It shows sinus tachycardia.

During the admission, renal function deteriorated, with serum creatinine rising above baseline levels (Table 6). This was accompanied by hypoalbuminemia, an elevated urine albumin-to-creatinine ratio, and heavy proteinuria on 24-hour urine collection. Renal ultrasonography demonstrated bilaterally enlarged kidneys.

**Table 6:** Laboratory investigations CRP: C-reactive protein; Hb: hemoglobin; LDH: lactate dehydrogenase

Laboratory Test	Result	Unit	Normal Values
Sodium	134	mmol/L	133–146
Potassium	3.9	mmol/L	3.5–5.3
Creatinine	85	µmol/L	53–97
Urea	4.0	mmol/L	2.5–7.8
CRP	12	mg/L	< 5
LDH	1633	IU/L	125–220
Repeat creatinine	107	µmol/L	53-97
24-hour urine protein	14.72	g/24Hour	0.00-0.15
Urine protein/ creatinine	364	mg/mmol cr	0 - 15

The availability of the previously pending autoimmune serology results, in conjunction with the patient's evolving clinical and laboratory findings, established the diagnosis of SLE. In addition to recurrent mAIHA, evidence of renal involvement was identified, characterized by impaired renal function, significant proteinuria, and hypoalbuminemia, consistent with lupus nephritis.

Diagnosis

The patient fulfilled the 2019 European League Against Rheumatism/American College of Rheumatology (EULAR/ACR) classification criteria for SLE [3]. The final diagnosis was newly diagnosed SLE presenting with recurrent mAIHA and lupus nephritis. The diagnosis was supported by positive ANA and anti-dsDNA antibodies (Table 4), progressive hemolytic anemia (Table 5), and biochemical and radiological evidence of renal involvement (Table 6).

Treatment During Second Admission

The patient was commenced on oral prednisolone 70 mg once daily, omeprazole 40 mg once daily, and folic acid 5 mg once daily. Intravenous paracetamol 1 g was prescribed as required for pyrexia and pain. She received one unit of PRBCs transfused through a blood warmer. Intravenous normal saline was administered for volume replacement in the setting of persistent diarrhea and vomiting.

Additional supportive management included calcium and vitamin D supplementation (Adcal-D3, one tablet daily). Venous thromboembolism prophylaxis was provided using graduated compression stockings, as the patient declined low-molecular-weight heparin administration. Empirical intravenous co-amoxiclav 1.2 g three times daily was initiated because of concerns regarding a possible underlying infection while microbiological investigations were pending.

Subsequent investigations failed to identify an infectious source, and the patient's fever was considered to be attributable to active SLE. Consequently, antimicrobial therapy was discontinued. During the course of admission, the patient developed evidence of lupus nephritis. As nephrology services were unavailable at our institution, a nephrologist was consulted for further evaluation and management.

Following the nephrology consultation, oral prednisolone therapy was escalated to intravenous methylprednisolone 250 mg once daily. The patient was subsequently referred to a tertiary renal centre for kidney biopsy. Histopathological examination predominantly demonstrated features of focal segmental glomerulosclerosis (FSGS). According to the biopsy report, the National Institutes of Health (NIH) activity score was 3/24, and the chronicity score was 1/12 [4]. These findings were consistent with relatively low levels of active inflammatory injury and minimal chronic irreversible damage.

A biopsy report was obtained from the GP record, as it was done in a tertiary care center. It showed the dominant abnormality to be FSGS with diffuse podocyte injury, extensive acute tubular injury, and features of heavy proteinuria. There was also immune-complex deposition with a full-house pattern, which was predominantly mesangial with focal subendothelial deposition and probable endocapillary hypercellularity in a rare segment.

In the given clinical context, the features just amounted to lupus nephritis International Society of Nephrology/Renal Pathology Society (ISN/PRS) class III (NIH activity score 3/24, chronicity score 1/12) with superimposed FSGS [5]. The presence of such diffuse podocyte injury and the observed tubular changes raised the possibility of background APOL1-related glomerular disease.

The patient was treated with intravenous belimumab (750 mg per infusion) in a tertiary care center. Concurrent immunosuppressive therapy included oral tablet mycophenolate mofetil 1 g twice daily and oral tablet prednisolone 30 mg once daily. A second belimumab infusion was administered two weeks after the initial dose during follow-up in the vasculitis clinic. GP records confirmed that the patient completed six cycles of belimumab therapy. Following treatment, she remained on maintenance therapy comprising oral tablet hydroxychloroquine 200 mg daily, oral tablet ramipril 5 mg daily, and calcium/vitamin D supplementation (calcium carbonate 1.25 g/cholecalciferol 400 IU, two tablets daily).

During follow-up, it was found that she was responding well to the treatment, with a decrease in proteinuria and normalization of hemoglobin and kidney function (Table 7). Her clinical symptoms also improved as her joint pain and swelling had ceased. Since the patient is no longer under our care, all the information was obtained from GP records, which may be incomplete. There were no complications noted that were a result of the treatment provided.

**Table 7:** Follow-up investigations Hb: hemoglobin

Laboratory Test	Result	Unit	Normal Values
Hb	125	g/l	115–165
Creatinine	57	µmol/l	53–97
Albumin	44	g/l	35-50
Urine albumin/creatinine ratio	89.8	(H)	0.0 - 3.5

## Discussion

SLE is a chronic multisystem inflammatory autoimmune disease of unknown etiology characterized by circulating autoantibodies and complex pathogenetic mechanisms [6]. Although SLE can occur at any age, it is rare before age 5 but most common in young people and predominantly affects females of reproductive age, particularly Europeans aged above 40 years. Age-standardized data from the UK reported an incidence rate of 8.3/100,000 for females and 1.4/100,000 for males, with a higher prevalence among Afro-Caribbean and South Asian people compared to Europeans [6]. The disease course is more severe in children and young adults, who may present with AIHA and lupus nephritis, as illustrated by the present case. The disease has relapsing and remitting characteristics that cause disease flares and cumulative organ damage, contributing to high morbidity. High mortality is primarily attributable to infections and cardiovascular events [6].

Causes and risk factors

The exact cause remains unknown; however, environmental agents in genetically susceptible individuals are thought to be fundamental [6-8]. The marked female predominance suggests that hormonal factors contribute to disease pathogenesis. Genetically, SLE is more prevalent in individuals who are HLA-positive for DR2, DR3, or DRB1 or have a defective C4 genetic component (C4A null allele) [7]. Environmental factors, including sunlight exposure, can exacerbate SLE rashes. Smoking is also considered a risk factor. Common causative medications include hydralazine, procainamide, isoniazid, methyldopa, phenytoin, carbamazepine, chlorpromazine, terbinafine, sulfasalazine, minocycline, and penicillamine [7,8]. In drug-induced SLE, anti-histone antibodies are positive, while anti-dsDNA and anti-Sm antibodies are negative [9].

Clinical features

SLE is a multisystem disease characterized by joint pain, joint swelling, and skin involvement, including a malar (butterfly) rash [8,10]. It can affect any organ system, including the respiratory system, e.g., serositis (pleurisy or pleural effusion), and the cardiovascular system, e.g., Libman-Sacks endocarditis and pericarditis. Some patients also present with unexplained fever, as in the present case. Patients may have central nervous system involvement, including cerebritis, seizures, and psychosis. Hematological manifestations include anemia, thrombocytopenia, and leukopenia. The most feared complication is lupus nephritis, which can lead to end-stage renal disease, requiring dialysis or kidney transplantation.

Diagnosis

Diagnosis requires a thorough history, physical examination, and targeted investigations to confirm the diagnosis. According to the 2019 EULAR/ACR classification criteria, a positive ANA is the entry criterion, followed by a weighted scoring system encompassing clinical and immunological domains [3]. The domains include constitutional, mucocutaneous, musculoskeletal, cardiovascular, renal, neuropsychiatric, hematological, respiratory, and immunological manifestations. At least one clinical criterion and a total score of 10 are required to establish the diagnosis. These criteria need not occur simultaneously, and only the highest-scoring criterion in each domain is counted. Moreover, ANA is positive in > 95% of SLE cases [3]. Negative results may occur in patients with anti-dsDNA, anti-Sm, anti-Ro, or anti-La antibodies that are not detected by the standard ANA test but still indicate SLE [10]. Anti-Sm antibodies are highly specific for SLE, whereas positive anti-dsDNA antibodies indicate disease activity [7,10]. Our patient fulfilled the classification criteria with a score of 34, which was well above the diagnostic threshold, even before renal biopsy results, which were later obtained through a tertiary care center.

Treatment

Treatment requires a multidisciplinary approach involving rheumatology, dermatology, hematology, and nephrology, among others, depending on the organs involved [8]. Lifestyle modifications, including skin protection and smoking cessation, are a crucial part of management. Acute flares are treated with corticosteroids, IV immunoglobulin, or immunological medications, whereas hydroxychloroquine is routinely used to reduce flares and improve survival [8].

This case presented a special challenge during blood transfusion, as identifying an exact blood match was challenging due to the presence of antibodies. Our patient presented with severe anemia during her first admission. At one point, she experienced dizziness and syncope due to the delay in blood transfusion. After receiving compatible blood, the patient was transfused blood using a blood warmer, followed by steroid therapy [11].

In patients with mAIHA, a blood warmer may be used during transfusion, particularly when clinically significant cold agglutinins are present, to minimize cold-induced red blood cell agglutination and hemolysis [11]. In AIHA, blood transfusion can worsen hemolysis because autoantibodies may attack donor RBCs. Therefore, corticosteroid therapy should be initiated to control hemolysis. Since autoantibodies complicate cross-matching, they often delay the availability of a compatible blood transfusion. If the patient is hemodynamically unstable and requires urgent transfusion, the least incompatible blood should be transfused. The transfusion should be administered slowly and closely monitored to minimize the risk of serious complications.

The subsequent development of lupus nephritis during the patient's second admission highlights the dynamic and potentially progressive nature of SLE. Lupus nephritis is one of the most serious complications of SLE and remains a major cause of long-term morbidity and mortality [12]. Early recognition and prompt immunosuppressive treatment are crucial to preserving renal function and preventing irreversible kidney damage. Because nephrology services were not available at our institution, transfer to a tertiary referral center was required for kidney biopsy, disease classification, and specialized management. The patient subsequently received combination therapy with corticosteroids, mycophenolate mofetil, hydroxychloroquine, and belimumab, reflecting current therapeutic strategies for significant renal involvement.

## Conclusions

mAIHA is a rare initial manifestation of SLE and may precede the development of other organ involvement, including lupus nephritis. This case highlights the importance of thoroughly investigating the underlying cause of mAIHA, particularly in young patients, to facilitate early recognition of systemic autoimmune disease. Prompt diagnosis, timely initiation of appropriate therapy, and multidisciplinary management are essential to improve clinical outcomes and reduce the risk of disease-related complications.
